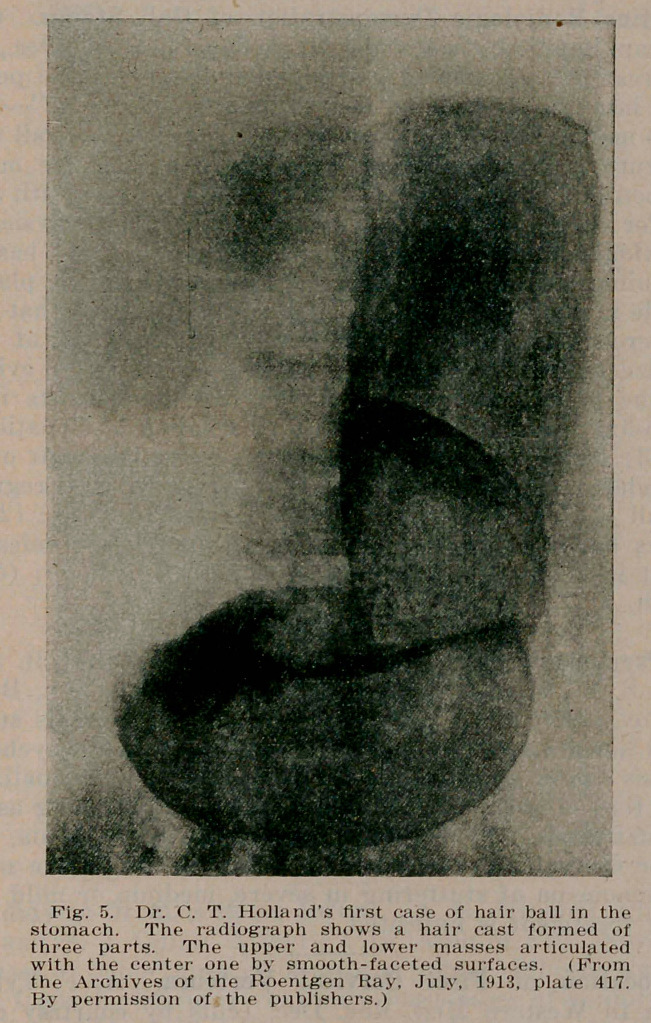# Hair Balls of the Stomach and Gastro-Intestinal Tract

**Published:** 1916-02

**Authors:** 


					﻿Hair Balls of the Stomach and Gastro-Intestinal Tract, Rudolph Matas, New Orleans, Surg. Gyn. & Obs., Nov. (Cuts by courtesy of editor). The first human case was that of Bau-damant, Jour, de Med. de Paris, 1779. References are given to collected series and to recent cases, including the present, bringing the total to 73. The first removed by operation was by Schoenborn 1883. The total operated upon is 44. 38 cases were gastrotomies, in several, including the present case, the hair ball reaching into the duodenum. 4 were enterotomies, one or more hair balls being removed from the intestine, mainly the ilium. 2 cases combined gastrotomy and enterotomy at different sittings. Hair balls are mainly due to habitual biting
and swallowing of the patient's own hair, and hence, 43 of lhe 44 operated cases occurred in girls and young women, the youngest patient being 6 years old, the oldest, a man, 52. In a few cases, other fibrous matter such as bits of string, wool, etc., are also swallowed as a habit. In only two of the 44 cases was the hair of a foreign nature, and in only one it was
not swallowed on account of a neurosis. Both of these were occupational cases, one in a woman engaged in spinning cow’s hair and who moistened it with her tongue, the other in a woman engaged in making bristle brushes, but the latter ate the bristles as a habit. (Note—We have frequently noted cases, mainly in women and children, in which the habit of biting the nails amounted to a neurosis, and included a perverted pleasure from actually swallowing the nails, even the
toe nails. We have never found reference to an accumulation thus caused. One of the nail-eaters who had heard of hair balls, thought that she had a tumor due to such an accumulation, but it proved to be merely the rectangular projection of the upper abdomen common in I host* with moderate anterior spinal curvature.)
In regard to, pre-operative diagnosis, aside from sex, history
of trichophagy and frequent finding of hair in stools or in wash water from the stomach, the author emphasizes the value of X-ray examinations. The article concludes with an essay on the ancient beliefs regarding bezoars
“The characteristic behavior of the greater curvatureonpal-pation, i.e., the greater curvature of the stomach becomes palpable when the stomach is contracted in active peristalsis and the bezoar is gripped firmly by the muscular contraction. It is not palpable when the stomach is relaxed and does not grip the foreign body.
The presence of fatty acid plates and needles in the contents of the fasting stomach, and the absence of starch and musclefibers. (These peculiar findings were not explained until the operation, when the hair ball was found to be infiltrated with fat which had resisted the digestive juices of the stomach and had been caught mechanically in the meshes, while the starchy
and albuminous substances were digested and discharged out of the stomach.)'’
If these preliminary facts can be obtained the pre-operative diagnosis only needs confirmation by the physical and radiographic examination which should show (a) a shadow in the region of the stomach (episgastrium) of a characteristic J-shape (Holland, 5) fringed at its lower border by a crescent lining formed by the gas-distended transverse colon. This will (b)localize the gastric area and exclude the spleen and omen-
tum, as these lie outside of the colonic loop. The liver can usually be distinguished from the stomach area by a faint darker outline. The further differentiation is accomplished by
4. Administration of a contrast meal of bismuth or barium in suspension, which will, at once, isolate the hair ball, either by surrounding it completely, leaving a paler and fainter area wherever the mixture has failed to penetrate the hair mass, or
in large hair balls, by the differentiating fluid finding its way between the stomach wall and the tumor. A distinct gastric contour is thus obtained within which the intragastric mass is defined by the gradual spreading of the contrast material on the outer surface of the hair ball. Or if this consists of two or more segments, as in Holland’s (5) first case, by the penetration of the contrast fluid into the interspace which subdivides the mass (see Fig. 5).
5. Finally, while the progress of the bismuth of barium meal is being watched, the displacement of the intragastric mass can be observed as it is moved upward or sideways by manipulation. If the displacement of a foreign body within the stom
ach can be made out clearly and unmistakably, and the mass is more or less molded on the contour of the stomach, it is almost impossible for the mass in the stomach to be anything else than a hair ball.
The real difficulty in the diagnosis of these cases lies in the usual inadvertence or unpreparedness of the operator for the possible presence of a hair ball.
				

## Figures and Tables

**Fig. 1. f1:**
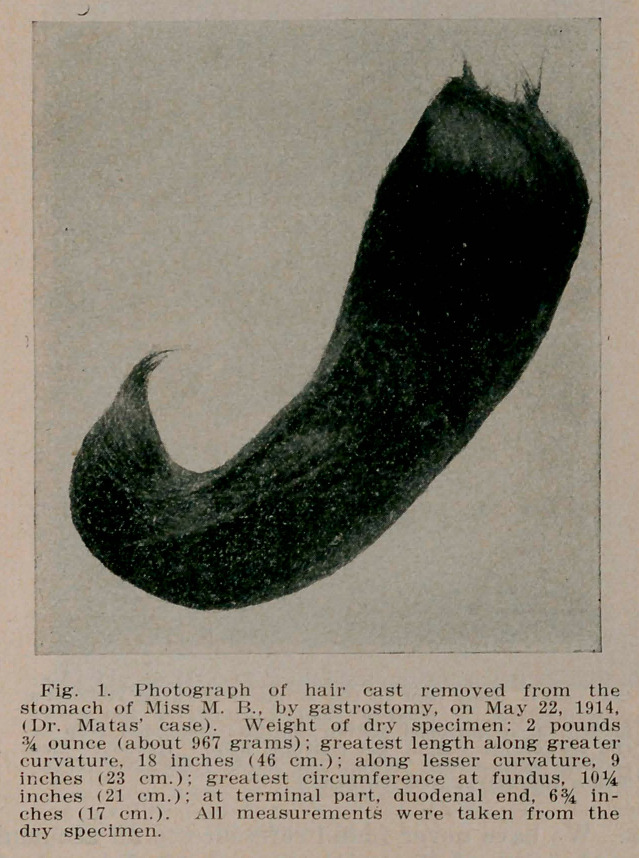


**Fig. 2. f2:**
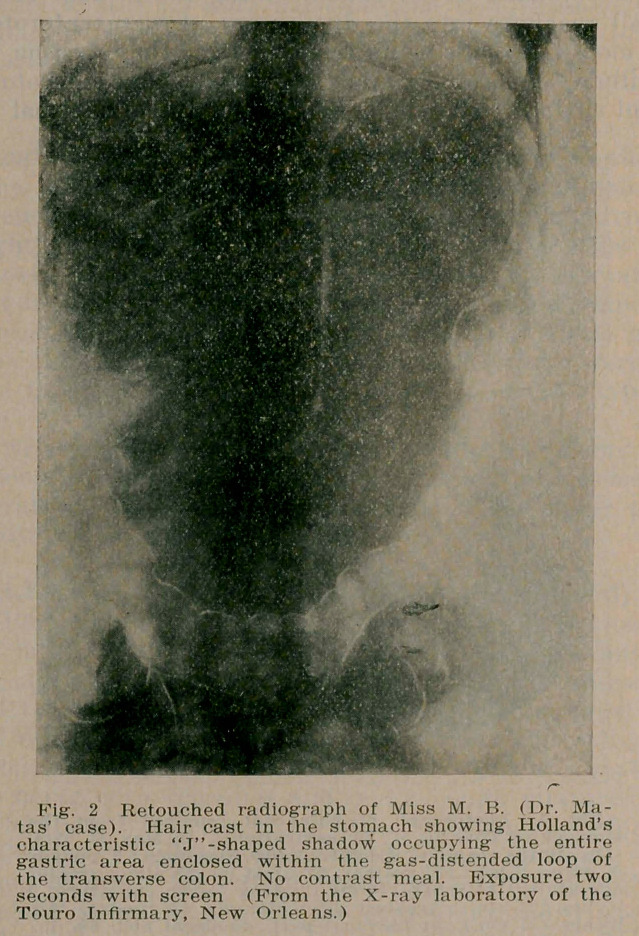


**Fig. 3. f3:**
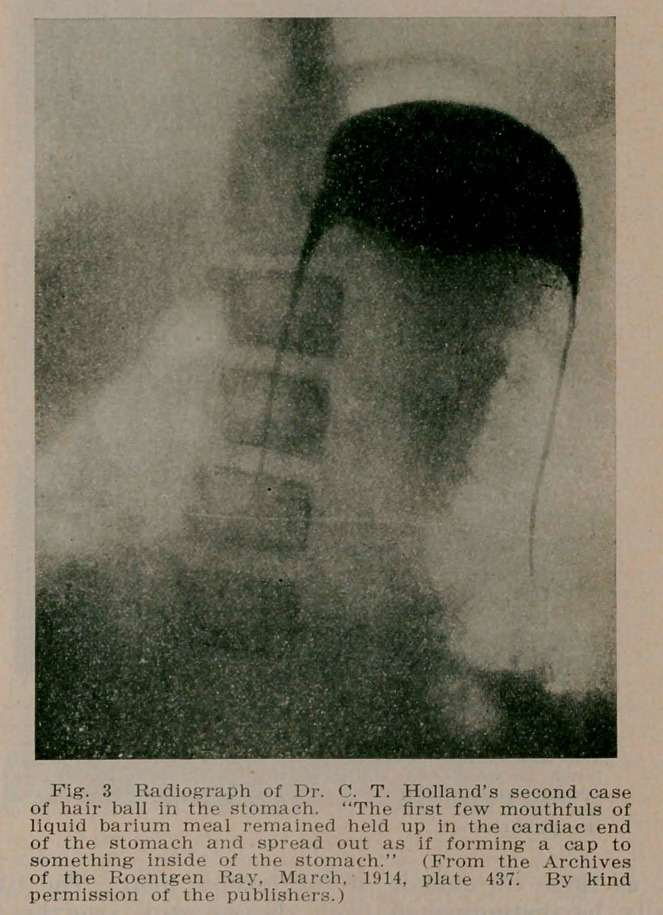


**Fig. 4. f4:**
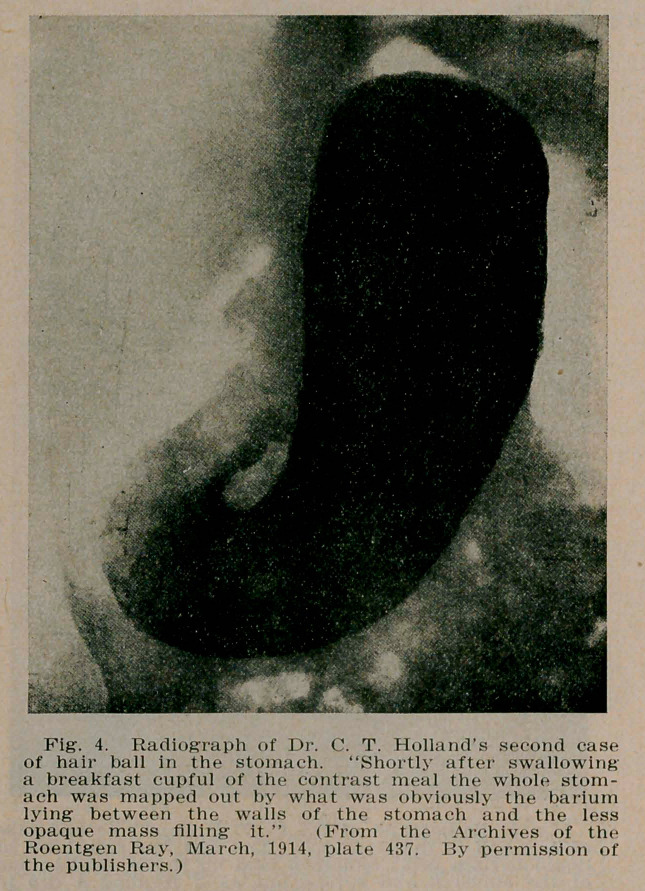


**Fig. 5. f5:**